# Analysis of the Primary Pathogenic Bacteria in Abscess Disease of Musk Deer Using Metagenomic Approaches

**DOI:** 10.3390/ani15081105

**Published:** 2025-04-11

**Authors:** Jingyao Hu, Xian An, Pengcheng Yang, Rongzeng Tan, Taoyue Chen, Jiatong Chen, Yifan Tao, Xuxin Li, Runbin Sun, Shouyun Zhang, Shuqiang Liu, Liangliang Yang

**Affiliations:** 1 College of Nature Conservation, Beijing Forestry University, Beijing 100107, China; 2 Zhangzhou Pien Tze Huang Pharmaceutical Co., Ltd., Zhangzhou 600436, China; 3 Huailai Zhiyangtianbao Technical Development Co., Ltd., Huailai, Zhangjiakou 075400, China; 4 Huailai County Forestry Bureau, Huailai, Zhangjiakou 075400, China; 5 Key Laboratory of Forest Protection of National Forestry and Grassland Administration, Research Institute of Forest Ecology, Environment and Protection, Chinese Academy of Forestry, Beijing 100091, China

**Keywords:** bacterial microbiota, abscess, musk deer, metagenomic sequencing

## Abstract

Abscesses are among the diseases affecting the survival of captive musk deer and are difficult to identify in their early stages. In this study, metagenomic sequencing, 16S rRNA sequencing, and paraffin sectioning were used to analyze the microbiota in the abscess musk deer pus group (AMP), abscess musk deer oral group (AMO), and healthy musk deer oral group (HMO) to compare the differences in microbiota in musk deer. By detecting differences in the oral microbiota through throat swabs, we aimed to monitor the early onset of abscess disease to facilitate timely intervention and treatment. The results showed that the alpha diversity of HMO microbiota was significantly higher than that of the AMP and AMO samples. Beta diversity results indicated that there were significant differences in the bacterial communities of HMO and AMO samples, and no significant difference was found between AMO and AMP samples. A taxonomic analysis of the bacterial species indicated that differences between HMO and AMP groups were found in the *Fusobacterium* and *Trueperella* species. *Fusobacterium* and *Trueperella* were the main pathogenic bacteria responsible for the occurrence of abscess diseases in forest musk deer in this study. Furthermore, the appearance of *Fusobacterium* and *Trueperella* in the oral cavity can serve as biomarkers for the early diagnosis of abscess disease in musk deer.

## Simple Summary

Captive forest musk deer are frequently afflicted by abscess disease, which is difficult to diagnose and treat in its early stages, often leading to high mortality rates. This study aimed to examine the differences in microbial communities between oral throat swabs and pus samples from musk deer, with the goal of enabling early detection and treatment of abscess disease through microbial profiling in the future. Samples were collected from three groups: oral throat swabs of healthy musk deer (HMO), oral throat swabs of diseased musk deer (AMO), and pus from abscesses (AMP). Metagenomic approaches and 16S rRNA sequencing were used to analyze and compare the microbial communities. The results revealed significant differences between the microbiota of healthy deer and diseased deer, while the microbial profiles of AMO and AMP were largely similar. Notably, *Fusobacterium* and *Trueperella* were detected in both AMO and AMP samples, suggesting that these bacteria are the primary pathogens responsible for abscess disease in forest musk deer. Based on these findings, we hope to develop a diagnostic throat swab test in the future that can detect early-stage abscess disease in musk deer, allowing for timely intervention and treatment.

## 1. Introduction

Forest musk deer (*Moschus berezovskii*) are small forest-dwelling animals. Due to habitat destruction and illegal poaching, the number of wild musk deer in China has decreased from 2.5 million in the 1950s to less than 100,000 [1]. China began to domesticate wild musk deer in the late 1950s. The initial goal was to reduce pressure on wild populations and provide resources for the recovery of these deer. The number of farmed musk deer in China exceeded 35,000 in 2012 [2,3]. The gradual increase in the number of these deer has laid a firm foundation for the restoration of wild populations. However, the captive musk deer population has long been affected by abscess diseases [4,5,6]. According to an investigation into this disease in musk deer in captivity, the number of deer suffering from abscesses was found to be more than 50% [7,8,9]. It has been observed that the presence of abscess disease is an important reason restricting the recovery of captive musk deer.

A few studies have shown that the occurrence of abscesses in mammals is closely related to multiple pathogenic microorganisms, including *Pseudomonas aeruginosa* [10], *Trueperella pyogenes* [11], *Klebsiella pneumoniae* [12], and other bacteria. These pathogens have also been found in cases of abscess disease in forest musk deer [13,14,15,16].

The abscesses occurring in musk deer can be divided into subcutaneous and visceral abscesses. Subcutaneous abscesses are mostly found in the jaw and eyes, as well as on the head and limbs [17]. Subcutaneous abscesses are difficult to spot under the cover of fur, while visceral abscesses are concentrated in the liver and lungs and are also difficult to detect externally. In the early stage of an abscess, there are no obvious changes in the behavior of musk deer, but in the later stages, low spirits and appetite are observed. Musk deer are timid and easily frightened, so their frequent capture and inspection is impractical. Collectively, these factors make it difficult to detect abscesses in their early stages in musk deer. Once subcutaneous abscesses appear visibly, or the deer begin displaying phenomena such as low spirits, the abscess disease is often already in a later stage and antibiotic treatment is no longer effective at this point. Therefore, there is an urgent requirement for the early detection of abscesses, especially visceral abscesses.

Throat swabs provide a simple and rapid method for detecting oral microbiota. The pharynx is the part of an animal which connects the mouth, nasopharynx, lower respiratory tract, and esophagus. It is open to the external environment via the mouth and nostrils. It is a major conduit through which many bacteria enter and invade the lower respiratory or digestive tracts and cause infection. The dynamic balance of the pharyngeal microenvironment is closely related to the occurrence and development of many animal diseases [18,19]. Therefore, a throat swab is of great significance in the early diagnosis of various visceral diseases, especially lung diseases.

In this study, the abscesses and throat swabs of captive deer with abscesses and the throat swabs of healthy musk deer were collected from Zhangjiakou, Hebei Province, China. We systematically studied the composition of pharyngeal microecology of the abscesses in forest musk deer using metagenomic and 16S rRNA sequencing, investigated the presence of abscess pathogens, and determined the main pathogenic bacteria causing abscesses. These methods can be used as an indicator for the detection of abscesses in musk deer, and will be helpful in further understanding the pathogenesis of forest musk deer abscesses and their diagnosis and treatment.

## 2. Materials and Methods

### 2.1. Sample Collection

The sample was collected from captive forest musk deer of Huailai Zhiyangtianbao Technical Development Co., Ltd., Zhangjiakou, Hebei, China.

Twenty-five forest musk deer were kept in one breeding house. The breeding house is divided into shelter areas and activity areas. The shelter area consists of six single rooms of about 3 m^2^, with concrete floors and good ventilation. The activity area is about 80 m^2^, with no shelter, and the ground is natural soil. Ear tags were used to identify individual musk deer. During November 2021, three musk deer showed symptoms of abscess successively in one breeding house. After the drainage of subcutaneous abscesses, pus and throat swabs were collected for the abscess musk deer pus group (AMP; *n* = 3) and abscess musk deer oral group (AMO; *n* = 3). Musk deer with abscess symptoms were isolated in one empty breeding house. By the end of December, all the musk deer with abscesses had died; we dissected them and collected the focal tissue. In January 2022, after ensuring the health of the remaining three forest musk deer in the original breeding house, we collected their throat swabs as the healthy musk deer oral group (HMO; *n* = 3). In January 2023, we collected throat swabs from an additional 10 healthy forest musk deer (HMO; *n* = 10) and from another 9 infected forest musk deer (AMO; *n* = 9).

Sterile disposable gloves were worn when collecting samples. The focal tissue was preserved with 4% paraformaldehyde. The pus and throat swab samples were placed in sterile centrifuge tubes immediately after collection and sealed to avoid cross-contamination between samples. All fresh samples were quick-frozen with liquid nitrogen and stored at −80 °C before further operation.

### 2.2. Pathological Section

After fixation with 4% paraformaldehyde for 48 h, the focal tissues were dehydrated with alcohol, made transparent with xylene, and embedded with paraffin. The embedded tissue was sliced into 5 μm slices, then sealed for observation after hematoxylin–eosin staining.

### 2.3. DNA Extraction, Library Construction, and Sequencing

The pus and throat swab samples in this study were extracted and sequenced by Beijing Biomarker Biotechnology Co., Ltd., Beijing, China. Library preparation was performed using the VAHTS^®^ Universal Plus DNA Library Prep Kit for Illumina (Vazyme Biotech, Nanjing, China, Cat# ND617-C3-02); the sequencing was conducted with the NovaSeq 6000 S4 Reagent Kit (Illumina, San Diego, CA, USA).

The DNA of samples was extracted using a DNA extraction kit (cetyltrimethylammonium bromide method). DNA levels were measured using a Qubit Fluorometer (Eppendorf, Hamburg, Germany). In metagenomic sequencing, after extracting the total DNA from the samples, primers were designed according to the ends of primers, and sequencing adapters were added to the ends of the primers. In 16S rRNA sequencing, design primers targeted the v3+v4 region of 16S rRNA (Table 1). The target sequences were amplified by PCR and the products purified, quantified, and homogenized to obtain a sequencing library. Metagenomic sequencing was performed on qualified libraries using the Illumina HiSeq platform (PE150/250) (Illumina, San Diego, CA, USA), while 16S rRNA sequencing was conducted on the Illumina NovaSeq 6000 (Illumina). The results were stored in FASTQ (referred to as fq) format, revealing the clean tag sequence. Bowtie2 was used to remove host contamination. Megahit software (v1.2.9) was used for sequence assembly, and QUAST (https://quast.sourceforge.net/) (quality assessment tool for genome assemblies) was used to evaluate the assembly results. MetaGeneMark (https://genemark.bme.gatech.edu/meta_gmhmmp.cgi, accessed on 8 April 2025) was used to identify the coding region in the genome by default parameters. MMseqs2 (https://mmseqs.com) was used to remove redundancy, the similarity threshold was set to 95%, and the coverage threshold was set to 90%.

### 2.4. Bioinformatics Analyses

Alpha diversity indices (i.e., ACE, Chao1, Shannon, and Simpson) were calculated using QIIME2 (https://qiime2.org) from rarefied samples and used to evaluate the richness and diversity indices of the bacterial community. Alpha diversity indices are presented as the means ± SD. The differences in alpha diversity indices and the relative abundance between groups of the top 10 phyla and genera were calculated using an independent-sample *t*-test (for the normally distributed data). Beta diversity was calculated using PCoA and PLS-DA, after which intra-group and inter-group beta distance boxplot diagrams were generated. A one-way analysis of similarity (ANOSIM) was performed to determine the differences in bacterial communities among groups. A *p*-value < 0.05 was considered statistically significant, and a *p*-value < 0.01 indicated that the differences were extremely significant.

The taxonomic annotation information of samples was obtained by BLAST (v2.2.26) in NR (Non-Redundant Protein Database). Species annotation results were obtained through the taxonomic information database in the NR library, and the abundance of species was calculated. The species abundances of each sample at the taxonomic levels of boundary, phylum, class, order, family, genus, and species were counted to construct the abundance table at the corresponding taxonomic level.

Linear discriminant analysis effect size (LefSe) analysis was performed to reveal the significant ranking of abundant modules in AMO and HMO, AMP, and AMO. A size–effect threshold of 3.5 on the logarithmic LDA score was used for discriminative functional biomarkers. The abundance of bacterial virulence factors in each group was statistically compared with the virulence factor database (VFDB) set A.

## 3. Results

### 3.1. Anatomy and Pathological Sections

**Figure 1:**
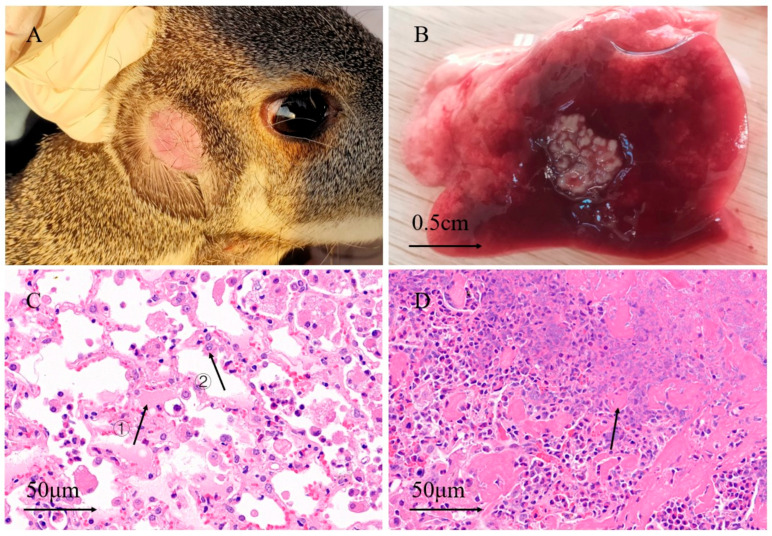
Lesions distribution and pathological section of musk deer abscess. (**A**) Facial abscess of musk deer after partial hair removal; (**B**) lung abscess stigmata; (**C**) lung paraffin section, arrow ①: tissue fluid in alveolar, arrow ②: inflammatory cell; (**D**) lung lesion tissue slice, arrow indicates pus.

Subcutaneous abscesses were found on both sides of the face and submaxillary of the sick musk deer, and were not obvious externally (Figure 1A). Visceral abscesses were concentrated in the lungs (Figure 1B), and a few lesions were spread throughout the chest. A pathological examination showed dilatation and hemorrhaging of the blood vessels, exudation of some tissue fluid, and the infiltration of many inflammatory cells in the alveolar wall (Figure 1C). The alveolar tissue near the lesion was filled with fluid, and the alveolar structure at the lesion site was obscured, as it contained a large quantity of pus (Figure 1D).

### 3.2. Raw Data Quality Control

Metagenomic sequencing was performed on the pus samples from diseased musk deer, as well as on throat swab samples from both healthy and diseased musk deer collected during the first sampling, and 2,466,201–4,746,220 different assembly sequences were detected in each sample (Table 2). In the assembly sequence, the content of G and C in the bases was greater than 41%, and the base with a base mass greater than or equal to 20 could reach 97.27%.

The 16S rRNA sequencing was performed on the throat swab samples from healthy and diseased musk deer collected during the second sampling. The raw reads ranged from 60,624 to 80,349, while the clean reads ranged from 60,448 to 80,120 (Table 3).

### 3.3. Biodiversity Composition Analysis Between Groups of Healthy and Abscessed Musk Deer

**Figure 2:**
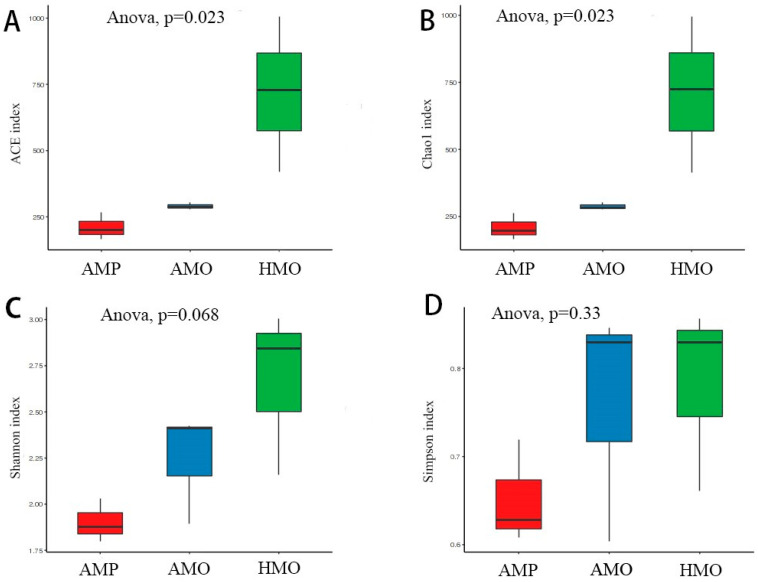
ACE, Chao 1, Shannon, and Simpson analyses of sick musk deer pus, sick musk deer throat swab, and healthy musk deer throat swab. Note: Boxplot of alpha diversity indices. Alpha diversity indexes are composite indexes reflecting abundance and consistency. (**A**) Ace and (**B**) Chao1 indices reflect species diversity at genus level in samples. (**C**) Shannon and (**D**) Simpson indices reflect species diversity at genus level in samples. The greater the Chao or ACE index, the higher the expected species richness of the microbiota; the smaller the Simpson index, the higher the diversity of the microbiota, and the greater the Shannon index, the higher the diversity of the microbiota (Student’s *t*-test).

The differences in species abundance and diversity among the AMP, AMO, and HMO groups were analyzed using the α diversity index (Shannon, Simpson, ACE, Chao 1). The ACE and Chao 1 results showed that the species diversity of HMO was significantly higher than those of AMP and AMO. That of AMO was also higher than that of AMP, but there was no significant difference (Figure 2A,B). There were no significant differences in the Shannon and Simpson indices (Figure 2C,D).

**Figure 3:**
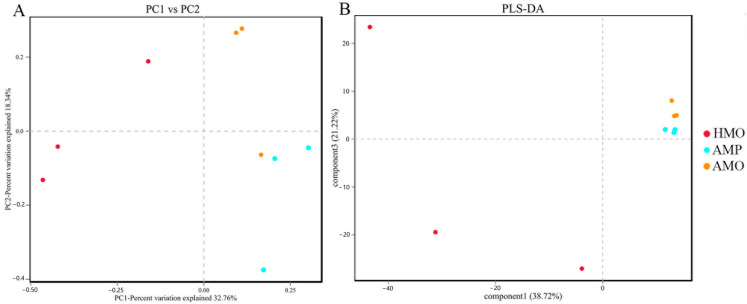
(**A**) PCoA analysis of sick musk deer pus, sick musk deer throat swab, and healthy musk deer throat swab. (**B**) PLS-DA analysis of sick musk deer pus, sick musk deer throat swab, and healthy musk deer throat swab. Note: Boxes represent the interquartile range (IQR) between first and third quartiles (25th and 75th percentiles, respectively), and the horizontal line inside the box defines the median. Whiskers represent lowest and highest values within 1.5 times the IQR from first and third quartiles, respectively.

The ß diversity indices PCoA and PLS-DA were used to analyze the differences in species diversity among AMP, AMO, and HMO groups (Figure 3). The results showed significant differences in bacterial communities between healthy and unhealthy musk deer. The clusters and samples were far apart, i.e., the HMO and AMO deer formed dissimilar and distinct structures of microbiota in the oral cavity (Figure 3A,B). Thus, the richness and evenness of the oral microorganisms of the musk deer populations were dissimilar. Meanwhile, within the AMO and AMP groups, the deer showed similar species composition, with samples being close together.

### 3.4. Species Annotation and Taxonomic Analysis

**Figure 4:**
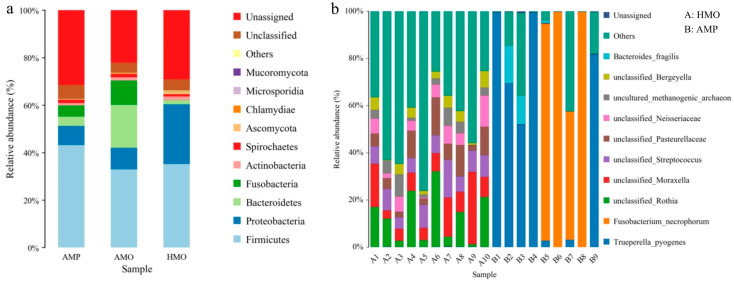
Species composition of pus, throat swabs from sick musk deer, and throat swabs from healthy musk deer. (**a**) Relative abundance of first experiment. (**b**) Relative abundance of second experiment. A represents HMO, B represents AMP. X-axis represents groups and y-axis represents relative abundance presented as a percentage. Only the top 10 species in abundance are shown in the figure; other species were combined as “Others”.

In both metagenomic sequencing and 16S rRNA sequencing, 33 phyla, 399 families, and 1073 species were detected. At the phyla level, AMP and AMO mainly included *Firmicutes*, *Proteobacteria*, *Fusobacteria*, *Bacteroidetes*, and *Actinobacteria*. HMO mainly included *Firmicutes*, *Proteobacteria*, *Bacteroidetes*, and *Actinobacteria* (Figure 4a). At the genus level, the microbial species detected by AMP mainly included *Trueperella*, *Fusobacterium*, and *Bacteroides*_FC4. Microbial species detected in HMO included *Streptococcus*, *Rothia*, *Moraxella*, *Bergeyella*, and *Bibersteinia* (Figure 4b). Compared with HMO, AMP also contained many other pathogenic microorganisms, such as *Fusobacterium* and *Trueperella*.

### 3.5. Annotation and Analysis of Virulence Factors

**Figure 5:**
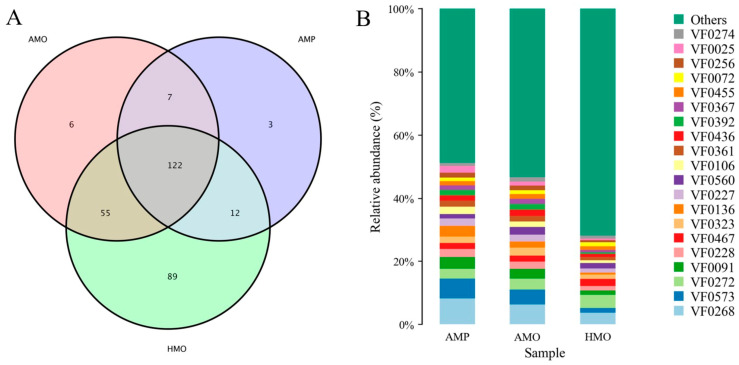
VFDB analysis. (**A**) Number of annotated virulence factors among different samples; (**B**) relative abundance of top 20 VF.

A total of 573 virulence factors (VF) were annotated through VFDB analysis. Among them, HMO contained the most virulence factors, AMO contained fewer, and AMP the least (Figure 5A). In AMP and AMO, the abundance of multiple virulence factors such as HitABC, Colibactin, Alginate, and Enterobactin was higher than in HMO (Figure 5B, Table 4). The functional annotation showed that most of the above virulence factors were related to the transport and binding of iron, DNA damage, and immune escape inhibition.

## 4. Discussion

Abscesses are among the most common diseases affecting the survival of captive musk deer, and early diagnosis is extremely challenging. This study utilized metagenomic sequencing technology and 16S rRNA sequencing technology to analyze the microbial communities in the pus samples of abscessed musk deer (AMP), the oral samples of abscessed musk deer (AMO), and the oral samples of healthy musk deer (HMO), comparing the differences in microbial communities within the deer. The aim was to detect differences in oral microbial communities through throat swabs, enabling the early identification of abscess disease for timely intervention and treatment.

The pathological examination showed obvious inflammatory cell infiltration in the lungs (Figure 1C,D). This finding of inflammatory cell infiltration is consistent with abscesses in musk deer from previous studies [17,20]. Clinically, abscesses are usually present in the hooves, lips, and face, and severe cases may infect the digestive tract, reproductive tract, liver, lungs, and other internal organs, resulting in the necrosis of some internal organs [21]. Musk deer abscess disease was initially determined according to the above clinical and histopathological symptoms.

Based on the results of ACE, Chao 1, Shannon, and Simpson indices, we believe that compared with the HMO, the species diversity of AMP and AMO was not significantly decreased, but the species abundance decreased significantly, a finding which may be related to the proliferation of pathogenic bacteria. Both the number and abundance of species present in the pus were low, which is similar to previous observations in humans [22,23], mice [24], and pigs [25]. A variety of studies have shown that the diversity and richness of oral microbial species play a key role in animal health, such as maintaining relevant functions and inhibiting the reproduction of pathogenic microorganisms [26]. Therefore, the diversity of oral flora in musk deer may be helpful for effective diagnoses. However, abscesses are caused by a variety of microorganisms, and the pathogenic bacteria causing abscess occurrence in captive musk deer may differ in various regions; therefore, further research is required to identify the bacteria at play.

The PCoA and PLS-DA analyses showed that the composition and relative abundance of the microflorae of AMO were significantly different from those of HMO. The microflorae of healthy deer individuals were more diverse and dispersed, while the microflorae on the throat swabs and in pus of sick deer were more similar. This was similar to findings regarding the phenomenon of bacterial aggregation in different diseases, as reported in previous studies [27,28,29]. This indicates that the microflorae of AMO and HMO have their own characteristics.

The species composition highlighted that the species showing significant differences between HMO and AMP was *Fusobacterium* in the first experiment, whereas the species exhibiting significant differences in the second experiment were *Fusobacterium* and *Trueperella*. The difference in the results of the two experiments may be due to the presence of different pathogenic bacteria in the two samples. *Fusobacterium* and *Trueperella* were the major pathogenic bacteria in the oral swabs and pus of sick deer, and different from those in the healthy deer. Therefore, *Fusobacterium* and *Trueperella* may be the main pathogenic bacteria causing abscesses in forest musk deer.

*Fusobacterium* causes infections in domesticated animals such as cattle [30] and sheep [31,32], and deer [33] and wild animals experience a common and high incidence of serious harm. The disease is often chronic and difficult to detect, and without timely treatment, the sick animals become severely emaciated, their health deteriorates, and some even die, with results similar to the symptoms of infections causing abscesses in musk deer [34,35]. *Fusobacterium* is the primary or secondary pathogen of many necrotic diseases, such as liver abscesses, rotting hoof, and laryngitis, found in animals [36,37] and is also known as the major pathogenic bacteria of human Lemierre’s syndrome, wherein the human infection manifests as pharyngitis, blood infection, and jugular vein thrombosis [38,39]. In our study, *Fusobacterium* was found in both the oral cavity and pus samples of individual musk deer, similar to abscesses caused by *Fusobacterium necrophorum* in other animals, suggesting that *Fusobacterium necrophorum* may play an important role as an opportunistic pathogen in the pathogenesis of abscesses in musk deer [40]. Our results also showed that *Trueperella* was detected in the pus from abscesses in infected deer, but not in the throat swab samples of healthy deer.

*Trueperella* is part of the upper respiratory and gastrointestinal skin and mucosal biota of an animal, and is also an opportunistic pathogen that can cause various purulent infections [41]. *Trueperella* has been detected in bovine purulent or caseous necrotizing pneumonia and other infectious lung diseases [42]. Clinical studies have shown an association between *Trueperella* and abscesses, pneumonia, and lymphadenitis [43]. In the present study, *Trueperella* was found in a single pus sample, indicating that *Trueperella* may play an important role as an opportunistic pathogen in the pathogenesis of abscesses found in musk deer, along with *Fusobacterium*.

Under normal circumstances, the oral bacteria maintain a dynamic balance, but when there is external stimulation or a decline in the body’s immunity or other conditions, the flora will change and oral bacteria will be found not only in the mouth, but will follow anatomical channels or enter the bloodstream to reach various parts of the body, resulting in the occurrence of infections [44,45]. Previous studies have shown that the mouth, as a reservoir of *Helicobacter pylor*, is inextricably linked with the development of stomach cancer [46]. It has been pointed out that oral microflorae are among the important reasons for oral complications in diabetic patients [47,48].

As per the results of the VFDB analysis, the top 20 most abundant virulence factors, such as *Escherichia coli* and *Staphylococcus aureus*, can be annotated as common opportunistic pathogens [49,50,51]. Due to the lack of research on the virulence factor types of *Fusobacterium* and *Trueperella*, in this study, only Lipopolysaccharide (LPS), a common virulence factor expressed in *Fusobacterium* and *Trueperella*, was detected [52]. This provides a certain reference for the role of the above-mentioned pathogens in the pathogenesis of forest musk deer. Other virulence factors, such as Capsule and Colibactin, were higher in prevalence in the samples from the sick forest deer compared to those from healthy deer. The functions of immune evasion and DNA damage may also cause diseases in musk deer, although further research is required to confirm this [53,54,55].

In the past, different scholars have often reported different abscess-causing pathogens in musk deer [13,14,15,16]. We found that *Escherichia coli*, *Pseudomonas aeruginosa*, and other pathogenic bacteria previously discovered by researchers also showed up in AMP and AMO. We believe there are three main reasons for this phenomenon. First of all, the previously studied musk deer samples came from the Sichuan and Shaanxi provinces of China, which are far away from Hebei province, the location of our sample. Abscess disease can be caused by a variety of pathogens; thus, the pathogens may be different in different environments. Secondly, in this study, the main site of visceral abscesses in forest musk deer was the lung, while previous studies showed that visceral abscesses were concentrated in the liver. This suggests that there may be different types of visceral abscesses, and therefore the pathogens causing different types of visceral abscesses may be different. Finally, previous studies mostly used basic microbiology experiments such as the spread plate method to isolate pathogenic bacteria, and this approach may miss some important pathogenic information, while metagenomics sequencing avoids omissions as much as possible. This also highlights the limitations of the experiment, as samples were only collected from the Huailai Musk Deer Farm, a single location, whereas the pathogenic bacteria may differ across farms.

Further studies are required to investigate whether additional pathogens may be involved and to elucidate the precise roles of different pathogens in the pathogenesis of extra-oral diseases (including respiratory and visceral manifestations). Moreover, more comprehensive research will be needed to accurately identify the causative agents of abscess disease. It is hoped that future research will be able to identify the pathogenic bacteria responsible for abscess disease and enable the early diagnosis of forest musk deer abscess disease through oral swabs, allowing for timely treatment.

## 5. Conclusions

In summary, *Fusobacterium* and *Trueperella* were first identified in pus and pharyngeal swabs from abscessed musk deer, and they may be important opportunistic bacteria that caused infections at the Zhangjiakou musk deer farm. Our study revealed that *Fusobacterium* and *Trueperella* may play a role in the development of visceral abscesses in forest musk deer.

In addition, the compositions of the pus and oral flora of musk deer with abscesses were very similar; thus, pharyngeal swabs may be used as a means of early diagnosis of abscesses in forest musk deer. The presence of *Fusobacterium* and *Trueperella* in the oral cavity can serve as a biomarker for the early diagnosis of abscess disease in musk deer, which is of great significance in the prevention and control of infections for breeding forest musk deer.

## Figures and Tables

**Table 1:** Primer sequence.

Name	Sequence (5′–3′)
F	ACTCCTACGGGAGGCAGCA
R	GGACTACHVGGGTWTCTAAT

**Table 2:** Evaluation statistics of sequencing data of each sample.

Sample ID	Clean Data Base (bp)	Number of Reads	GC (%)	Q20 (%)	Q30 (%)
AMP1	1,037,601,201	3,399,457	43.24	96.84	92.55
AMP2	1,103,460,085	3,593,644	45.26	96.25	91.46
AMP3	1,024,944,773	3,340,425	44.52	96.45	91.79
AMO1	1,216,288,821	3,978,145	42.94	96.94	92.53
AMO2	1,215,037,665	3,974,587	42.49	97.13	92.9
AMO3	841,226,856	2,708,702	46.57	96.43	91.88
HMO4	769,195,329	2,466,201	43.65	96.24	91.48
HMO5	1,440,773,547	4,732,485	41.62	96.95	92.43
HMO6	1,444,528,949	4,746,220	43.95	97.27	93.23

Note: Clean Data Base: Counts of clean reads (post quality control and assembly); Number of Reads: Counts of effective reads after chimeric reads removal; GC (%): GC content, i.e., proportion of G and C in all bases; Q20 (%): Percentage of bases with Q-score larger or equal to Q20; Q30 (%): Percentage of bases with Q-score larger or equal to Q30.

**Table 3:** Evaluation statistics of second sample sequencing data.

**Sample ID**	**Raw Reads**	**Clean Reads**	**Denoised Reads**	**Merged Reads**	**Nonchimeric Reads**
HMO4	79,834	79,572	78,409	77,227	71,391
HMO5	80,328	80,073	79,386	78,940	75,743
HMO6	80,156	79,857	78,035	76,232	70,728
HMO7	79,927	79,651	77,793	75,756	70,498
HMO8	80,051	79,801	78,544	76,761	71,292
HMO9	80,131	79,858	78,184	76,142	71,194
HMO10	79,981	79,731	79,048	78,515	72,828
HMO11	80,035	79,762	78,962	78,601	72,107
HMO12	79,940	79,664	78,201	76,634	68,675
HMO13	79,909	79,658	79,067	78,758	75,252
AMO4	80,349	80,120	79,813	79,704	79,685
AMO5	79,953	79,704	78,430	77,336	75,599
AMO6	80,018	79,762	78,012	76,522	73,992
AMO7	80,083	79,842	79,563	79,474	79,458
AMO8	60,624	60,448	60,057	59,898	58,629
AMO9	79,429	79,169	78,982	78,934	77,530
AMO10	79,812	79,591	79,284	78,872	67,972
AMO11	79,944	79,698	79,491	79,429	78,005
AMO12	79,740	79,497	77,661	75,727	75,780

Note: Raw Reads: Sequencing reads obtained directly from sequencer; Clean Reads: Counts of clean reads (post quality control and assembly); Denoised Reads: Number of reads remaining after noise removal from Clean Reads; Merged Reads: Count of sequences generated by overlapping and merging Denoised Reads; Nonchimeric Reads: Final number of sequences after chimeric read removal.

**Table 4:** Note information on virulence factors of top 20% relative abundance.

ID	VF_Name	VF_Function
VF0268 *	HitABC	HitABC(fbpABC) operon
VF0573 *	Colibactin	Inducing DNA damage
VF0272	FbpABC	periplasmic Fe3+ binding protein
VF0091 *	Alginate	Forming biofilm
VF0228 *	Enterobactin	Iron uptake
VF0467	Acinetobactin	High-affinity catechol-hydroxamate siderophore
VF0323 *	Capsule	Bacterial survival, persistance, evasion of host immune response
VF0136 *	Yersiniabactin	FyuA/Psn-Irp system uses yersiniabactin
VF0227 *	Chu	Iron uptake
VF0560	Capsule	Assisting in evading the host immune system
VF0106 *	MgtBC	Intracellular survival or for virulence
VF0361 *	Capsule	Contributes to host immune evasion
VF0436 *	Capsule I	Key virulence determinant
VF0392 *	O-antigen	LPS O antigen mutants
VF0367 *	LPS	Early survival inside macrophage
VF0455 *	MntABC	Quenches ROS
VF0072	ClpC	ATPase promoting early escape
VF0256 *	Shu	Shu heme utilization locus
VF0025 *	FHA	Adherence to ciliated respiratory epithelial cells
VF0274	Capsule	Inhibits the complement factor C3b
Others		

Note: VF marked * represents relative abundances in AMP and AMO higher than those in HMO.

## Data Availability

The data that support the findings of this study are available on request from the corresponding author, S.L., upon reasonable request.

## References

[1] Wu J.Y., Wang W. (2006). The Musk Deer of China.

[2] Li L.H., Huang X.Y., Liu G., Wang W.X., Wei N., Liu Y., Hu D.F., Meng M. (2012). The Status of Captive Population of Musk Deer and Analysis of its Farming Development in China. J. Sichuan J. Zool..

[3] He L., Li L.H., Wang W.X., Liu G., Liu S.Q., Liu W.H., Meng M., Hu D.F. (2014). Discussion about relationship between biological characters and farming development of musk deer. For. Resour. Manage.

[4] Li L., Wang B.B., Ge Y.F., Wan Q.H. (2014). Major histocompatibility complex class II polymorphisms in forest musk deer (*Moschus berezovskii*) and their probable association with purulent disease. Int. J. Immunogenetics.

[5] Yan M., Yan Q.G., Yang G.Y. (2016). The mass diseases of captive musk deer. J. Econ. Anim..

[6] Li Y.M., Hu X.L., Yang S., Zhou J.T., Qi L., Sun X., Fan M., Xu S., Cha M., Zhang M. (2018). Comparison Between the Fecal Bacterial Microbiota of Healthy and Diarrheic Captive Musk Deer. Front. Microbiol..

[7] Wang J.L., Zhang T.Q., Zhu C.H.S. (1990). Preliminary analysis on the death of forest musk deer. J. Anim. Husb. Vet. Med..

[8] Zhao K.L., Li X.X., Pa P., Zeng B., Zhang X.Y., Yue B.S. (2011). Isolation and identification on Pathogens of Musk Deer Abscess Disease and Antibiotic Susceptibility Assay. Sichuan Anim..

[9] Zhao K.L., Liu Y., Zhang X.Y., Paha’erding Palahati Wang H.N., Yue B.S. (2011). Detection and characterization of antibiotic-resistance genes in Arcanobacterium pyogenes strains from abscesses of forest musk deer. J. Med. Microbiol..

[10] Heimesaat M.M., Escher U., Grunau A., Kühl A.A., Bereswill S. (2019). Multidrug-Resistant Pseudomonas aeruginosa Accelerate Intestinal, Extra-Intestinal, and Systemic Inflammatory Responses in Human Microbiota-Associated Mice with Subacute Ileitis. Front. Immunol..

[11] Cohen B.S., Belser E.H., Killmaster C.H., Bowers J.W., Irwin B.J., Yabsley M.J., Miller K.V. (2015). Epizootiology of cranial abscess disease in white-tailed deer. (*Odocoileus virginianus*) of Georgia, USA. J. Wildl. Dis..

[12] Joob B., Wiwanitkit V. (2017). Klebsiella pneumoniae Invasive Liver Abscess Syndrome and Endophthalmitis. J. Emerg. Med..

[13] Luo Y., Cheng J.G., Li Q.B., Cai Y.H., Jia L., Zhao C. (2006). Isolation and identification of the pathogen of *Escherichia coli* suppurosis in musk deer. J. Heilongjiang Anim. Sci. Vet. Med..

[14] Li Q.B., Yan Q.G., Kang J.P., Li P., Xiong W.P. (2012). Isolation and Identification of Purulent Bacteria from Forest Musk Deer (*Moschus berezovskii*). J. BBC Wildl. Mag..

[15] Tang J., Hu H., Wang Y.Q., Li F.R., Liu W.H. (2011). Isolation and Identification of Corynebacterium Pyogenes from Forest Musk Deer. J. China Herbiv..

[16] Xi L.X., Chen Z.R., Song H.Y., Ren L., Wen J.F., Huang J., Yang R., Yang G.Y., Wang H.Y., Yan Q.G. (2018). Identification and drug-resistance, pathogeny characteristics Pseudomonas aeruginosa from captive forest musk deer. J. Microbiol..

[17] Wu S.Z., Yang L.F., Qian L.M., Yang M.H., Li Q.H., Chen P.F. (2021). Isolation and Identification of Pathogenic Bacteria for Abscess in Captive Forest Musk Deer (*Moschus berezovskii*). Chin. J. Wildl..

[18] Yeoh Y.K., Chan M.H., Chen Z., Lam E.W.H., Wong P.Y., Ngai C.M., Chan P.K.S., Hui M. (2019). The human oral cavity microbiota composition during acute tonsillitis: A cross-sectional survey. BMC Oral Health.

[19] Kilian M., Chapple I.L., Hannig M., Marsh P.D., Meuric V., Pedersen A.M.L., Tonetti M.S., Wade W.G., Zaura E. (2016). The oral microbiome—An update for oral healthcare professionals. Br. Dent. J..

[20] Pan S.H., Wan Y.Y., Zhang K.B. (2002). Preliminary report on etiological diagnosis of subcutaneous and lymphatic abscess in goats. Guizhou Agric. Sci..

[21] Pillai D.K., Amachawadi R.G., Baca G., Narayanan S.K., Nagaraja T.G. (2021). Leukotoxin production by *Fusobacterium necrophorum* strains in relation to severity of liver abscesses in cattle. Anaerobe.

[22] Jensen J.L., Bergem H.O., Gilboe I.M., Husby G., Axéll T. (1999). Oral and ocular sicca symptoms and findings are prevalent in systemic lupus erythematosus. J. Oral Pathol. Med..

[23] Wade W.G. (2013). The oral microbiome in health and disease. Pharmacol. Res..

[24] Kobayashi R., Ogawa Y., Hashizume-Takizawa T., Kurita-Ochiai T. (2020). Oral bacteria affect the gut microbiome and intestinal immunity. Pathog. Dis..

[25] Li Y.T., Li J.L., Yang S.H., Xia D.S., Zhang C.M., Wang S.L. (2006). Comparative study on oral floras of minipigs, rats and human being. Beijing J. Stomatol..

[26] Jiang F., Song P., Wang H., Zhang J., Liu D., Cai Z., Gao H., Chi X., Zhang T. (2022). Comparative analysis of gut microbial composition and potential functions in captive forest and alpine musk deer. Appl. Microbiol. Biotechnol..

[27] Li Y.M., Hu X.L., Yang S., Zhou J.T., Zhang T.X., Qi L., Sun X., Fan M., Xu S., Cha M. (2017). Comparative Analysis of the Gut Microbiota Composition between Captive and Wild Forest Musk Deer. Front. Microbiol..

[28] Sims T.T., Colbert L.E., Zheng J., Delgado Medrano A.Y., Hoffman K.L., Ramondetta L., Jazaeri A., Jhingran A., Schmeler K.M., Daniel C.R. (2019). Gut microbial diversity and genus-level differences identified in cervical cancer patients versus healthy controls. Gynecol. Oncol..

[29] Rullo J., Far P.M., Quinn M., Sharma N., Bae S., Irrcher I., Sharma S. (2020). Local oral and nasal microbiome diversity in age-related macular degeneration. Sci. Rep..

[30] Tadepalli S., Narayanan S.K., Stewart G.C., Chengappa M.M., Nagaraja T.G. (2009). *Fusobacterium necrophorum*: A ruminal bacterium that invades liver to cause abscesses in cattle. Anaerobe.

[31] Shanthalingam S., Narayanan S., Batra S.A., Jegarubee B., Srikumaran S. (2016). *Fusobacterium necrophorum* in North American Bighorn Sheep (*Ovis canadensis*) Pneumonia. J. Wildl. Dis..

[32] Farooq S., Wani S.A., Hassan M.N., Aalamgeer S., Kashoo Z.A., Magray S.N., Bhat M. (2018). The detection and prevalence of leukotoxin gene variant strains of Fusobacterium necrophorum in footrot lesions of sheep in Kashmir, India. Anaerobe.

[33] Brooks J.W., Kumar A., Narayanan S., Myers S., Brown K., Nagaraja T.G., Jayarao B.M. (2014). Characterization of Fusobacterium isolates from the respiratory tract of white-tailed deer (*Odocoileus virginianus*). J. Vet. Diagn. Investig..

[34] Wang K.J., Zhang H.Y., Chen L.Z., Liu X.Y., Cheng S.P., Xu M., Miao L.G., Zhang H.T., Qian G.C., Yang F.H. (2000). Isolation and Identification of the Causative agent of Necrobacillosis in Deer. Chin. J. Prev. Vet. Med..

[35] Kjærulff A.M., Thomsen M.K., Ovesen T., Klug T.E. (2015). Clinical and biochemical characteristics of patients with *Fusobacterium necrophorum*-positive acute tonsillitis. Eur. Arch. Oto-Rhino-Laryngol..

[36] Narayanan S.K., Nagaraja T.G., Chengappa M.M., Stewart G.C. (2002). Leukotoxins of gram-negative bacteria. Vet. Microbiol..

[37] Batty A., Wren M.W., Gal M. (2005). Fusobacterium necrophorum as the cause of recurrent sore throat: Comparison of isolates from persistent sore throat syndrome and Lemierre’s disease. J. Infect..

[38] Alfreijat M. (2016). A case of I emierre’s Syndrome with a brief literature review. J. Infect. Public Health.

[39] Riordan T. (2007). Human infection with *Fusobacterium necrophorum* (Necrobacillosis), with a focus on Lemierre’s syndrome. Clin. Microbiol. Rev..

[40] Wang Q.J., Li W.X., Jin H., Zhang X.Y., Cai S., Jian Y.N., Xu L., Li X.P., Chao S.Y., Ma L.Q. (2017). Diagnose of pathogens on liver abscess from lamb in Haixi area of QingHai Province. Chin. Qinghai J. Anim. Vet. Sci..

[41] Rzewuska M., Kwiecień E., Chrobak-Chmiel D., Kizerwetter-Świda M., Stefańska I., Gieryńska M. (2019). Pathogenicity and Virulence of Trueperella pyogenes: A Review. Int. J. Mol. Sci..

[42] Zhou Y., Shao Z., Dai G., Li X., Xiang Y., Jiang S., Zhang Z., Ren Y., Zhu Z., Fan C. (2023). Pathogenic infection characteristics and risk factors for bovine respiratory disease complex based on the detection of lung pathogens in dead cattle in Northeast China. J. Dairy Sci..

[43] Ribeiro M.G., Risseti R.M., Bolaños C.A., Caffaro K.A., de Morais A.C., Lara G.H., Zamprogna T.O., Paes A.C., Listoni F.J., Franco M.M. (2015). Trueperella pyogenes multispecies infections in domestic animals: A retrospective study of 144 cases (2002 to 2012). Vet. Q..

[44] Basis C.M., Erb-Downward J.R., Dickson R.P., Freeman C.M., Schmidt T.M., Young V.B., Beck J.M., Curtis J.L., Huffnagle G.B. (2015). Analysis of the upper respiratory tract microbiotas as the source of the lung and gastric microbiotas in healthy individuals. Mbio.

[45] Wassenaar T.M., Juncos V.A., Zimmermann K. (2021). Interactions between the Gut Microbiome, Lung Conditions, and Coronary Heart Disease and How Probiotics Affect These. Int. J. Mol. Sci..

[46] Sun J.H., Ying J., Li X.L., Hou B.X., Zhang Z.T. (2018). Characteristics of oral microbiome in gastric cancer patients. J. Beijing J. Stomatol. Beijing J. Stom..

[47] Liu B., Zhang A.H., Zhang Z. (2017). Study on the Relationship between UCA1, Nuclear Factor, Oral Flora and the Occurrence and Development of Oral Cancer. J. Pract. J. Cancer.

[48] Yang J.J., Zhang J.M., Chen B., Meng Q.S., Shi F., Shi G.S., Zhang L. (2017). Analysis of the oral microbiota and its potential association with gastric cancer. J. Pathog. Biol..

[49] Plata M.R., Contento A.M., Villaseñor M.J., Cabezas M.L., Ríos A. (2009). Development of a novel biotoxicity screening assay for analytical use. Chemosphere.

[50] Pakbin B., Brück W.M., Rossen J.W.A. (2021). Virulence Factors of Enteric Pathogenic Escherichia coli: A Review. Int. J. Mol. Sci..

[51] Veetilvalappil V.V., Manuel A., Aranjani J.M., Tawale R., Koteshwara A. (2022). Pathogenic arsenal of Pseudomonas aeruginosa: An update on virulence factors. Future Microbiol..

[52] Holt S.C., Kesavalu L., Walker S., Genco C.A. (1999). Virulence factors of Porphyromonas gingivalis. Periodontology 2000.

[53] Makino S., Watarai M., Cheun H.I., Shirahata T., Uchida I. (2002). Effect of the lower molecular capsule released from the cell surface of Bacillus anthracis on the pathogenesis of anthrax. J. Infect. Dis..

[54] Vecchiarelli A.G., Havey J.C., Ing L.L., Wong E.O., Waples W.G., Funnell B.E. (2013). Dissection of the ATPase active site of P1 ParA reveals multiple active forms essential for plasmid partition. J. Biol. Chem..

[55] Strakova N., Korena K., Karpiskova R. (2021). Klebsiella pneumoniae producing bacterial toxin colibactin as a risk of colorectal cancer development—A systematic review. Toxicon.

